# Multidisciplinary Intersections on Artificial-Human Vividness: Phenomenology, Representation, and the Brain

**DOI:** 10.3390/brainsci12111495

**Published:** 2022-11-03

**Authors:** Violetta Molokopoy, Amedeo D’Angiulli

**Affiliations:** 1 Department of Neuroscience, Carleton University, Ottawa, ON K1S 5B6, Canada; 2 NICER Lab, Carleton University, Ottawa, ON K1S 5B6, Canada

**Keywords:** vividness, vivid knowledge representations, artificial neural networks, consciousness, phenomenology

## Abstract

This article will explore the expressivity and tractability of vividness, as viewed from the interdisciplinary perspective of the cognitive sciences, including the sub-disciplines of artificial intelligence, cognitive psychology, neuroscience, and phenomenology. Following the precursor work by Benussi in experimental phenomenology, seminal papers by David Marks in psychology and, later, Hector Levesque in computer science, a substantial part of the discussion has been around a symbolic approach to the concept of vividness. At the same time, a similar concept linked to semantic memory, imagery, and mental models has had a long history in cognitive psychology, with new emerging links to cognitive neuroscience. More recently, there is a push towards neural-symbolic representations which allows room for the integration of brain models of vividness to a symbolic concept of vividness. Such works lead to question the phenomenology of vividness in the context of consciousness, and the related ethical concerns. The purpose of this paper is to review the state of the art, advances, and further potential developments of artificial-human vividness while laying the ground for a shared conceptual platform for dialogue, communication, and debate across all the relevant sub-disciplines. Within such context, an important goal of the paper is to define the crucial role of vividness in grounding simulation and modeling within the psychology (and neuroscience) of human reasoning.

## 1. Varieties of Vividness and Their Place in Science

**Figure 1:**
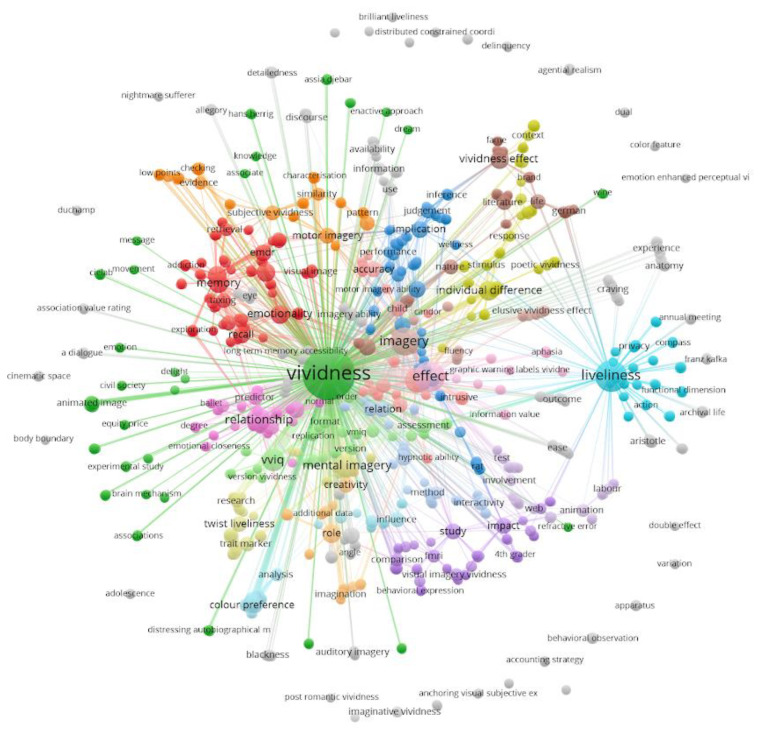
Network of words associated with ‘vividness’ or ‘liveliness’ in the titles of research articles. The network exposes how currently ‘vividness’ and ‘liveliness’ are potentially regarded as two distinct concepts or are researched independently by different disciplines. Figure taken from [1].

In this scoping review, we examine the multifaceted concept of vividness given in the literature during the last fifty years, highlighting that properties and definitions of the construct rarely overlap even when they seem to refer to very similar latent constructs. The review contrasts the different and linked usage of vividness in each of the scientific disciplines (see Figure 1) considered and aims to provide a common conceptual framework for researchers on vividness in different fields to communicate with each other.

The concept is first dissected as it was initially raised in computer science and artificial intelligence, then backtracked to its original development in cognitive psychology (specifically in the realm of mental imagery), until the current revivals in brain-inspired AI and robotics. The main dividing line appears to be the ubiquitous status of the vividness concept, from being essentially a technical device (a constraint to rendering sentence reasoning tractable) to alternative forms of analysis of psychological and brain processes (see Figure 1). An in-depth review of the ongoing transdisciplinary research leads to a proposed integration of the different viewpoints according to a brain-inspired model usable by artificial intelligence research on consciousness. This proposal is based on key phenomenological principles and concepts such as intentionality. We argue that unification of different fields on vividness is not only timely but urgent given the rapid development of AI technology, and possible missed opportunities for contributing across a wide range of disciplines within science. 

## 2. Vividness in Artificial Intelligence

Expressiveness and tractability in knowledge representation have been at the center of parallel traditions in artificial and human reasoning for over fifty years now. In computer science, following the seminal paper by Hector Levesque [2], a substantial part of the discussion, research, and advances have evolved around the concept of vivid knowledge. At the same time, a similar concept, vividness, linked to semantic memory, imagery, and mental models, has had a long history in cognitive psychology, with new emerging links to cognitive neuroscience. However, it is not clear what has been of the concept of vividness in cognitive science. One would imagine that it might have evolved and be embedded somehow in useful forms across disciplines. 

In 2008, one of the present authors asked Levesque about the state of vividness in cognitive science [3]. His response was the following:

*“Not much of interest in my version of vividness in the Cog Sci community to my knowledge. What mainly happened after a paper I wrote in 1986 ("Making believers out of computers") is that people in [knowledge representation] took it on as a simple constraint on how reasoning with sentences could be made computationally feasible. Until that point, reasoning with large collections of sentences looked quite impractical computationally, and even undecidable in some cases. (That my version of vividness happened to coincide with some constraints on psych plausibility and imagery was viewed as a bonus.) The bulk of the work since then builds on and extends the notion of vividness in various ways: how can you keep reasoning with sentences feasible while generalizing the scope of sentences allowed to account for ever richer forms of reasoning. All the work I know in this vein is pretty technical but "levesque vivid reasoning" gets a lot of hits in Google!”*

In his response, Levesque denoted that the interest in vividness, as defined in his 1986 “Making believers out of Computers” [2], was of a technical nature, and not as much one explored in cognitive science. There was an interest in rendering sentence reasoning tractable, and Levesque’s definition of vividness helped inform some of the constraints that researchers faced at the time. Since then, sentence reasoning tractability continues to be expanded to allow for more complex forms of reasoning, but such investment and research in vividness as an alternative form of reasoning is still in the early stages [3].

Levesque’s response sketches out the history of this topical concept, but it also manifests the need to unify the concept of vividness under one roof, to explore how the concept of vivid representing can facilitate complex forms of reasoning, and what constraints it can help navigate when used in conjunction with other forms of reasoning. 

The purpose of this paper is to explore the state of the art, advances, and further potential developments in vivid knowledge representation while laying the ground for a common conceptual platform for dialogue, communication, and debate across all the relevant sub-disciplines. Within such context, an important goal of this paper is to define the importance of vividness for simulation and modeling and how it is based and intrinsically linked to some dimensions of human psychology and neurophysiology. 

In this paper, first, we will briefly review most current research developments on vividness in computer science and symbolic artificial intelligence. Then, we will briefly review vividness research in the cognitive psychology of visual mental imagery. We then review vividness in brain-inspired AI. Finally, we will attempt to offer a synthesis of all these types of vividness that integrates in one all the different perspectives, and which offers a direct account of why we need and use visualization, simulation, and modeling as a way of an internal and external (public) tool for reasoning. We end the paper with relevant considerations about ethical future concerns which are implied by the new emerging brave field of AI consciousness derived from the research field on “synthetic phenomenology”, that is, “the attempt to use the states, interactions and capacities of an artificial agent for the purpose of specifying the contents of conscious experience” [4,5].

### 2.1. Vividness in Symbolic AI

#### Levesque’s Vivid Knowledge Bases

Humans as well as computers have great difficulty working with logic. This is why, at a first glance, incorporating logic computations to the study of cognition may appear intractable [6]. Levesque suggests that logic should be considered as computationally complex, such that using it in some cognitive tasks may be easy while hard in others. In fact, it has been shown that even very small changes could cause a manageable problem to become NP-hard, such that it requires polynomial time to be solved [2]. Thus, to ensure tractability in information processing tasks, the deviation from classical logic should be in direct correspondence to the deviation needed for psychological plausibility [6]. This leads Levesque to suggest that in order to maintain a problem tractable some restrictions should apply. 

**Figure 2:**
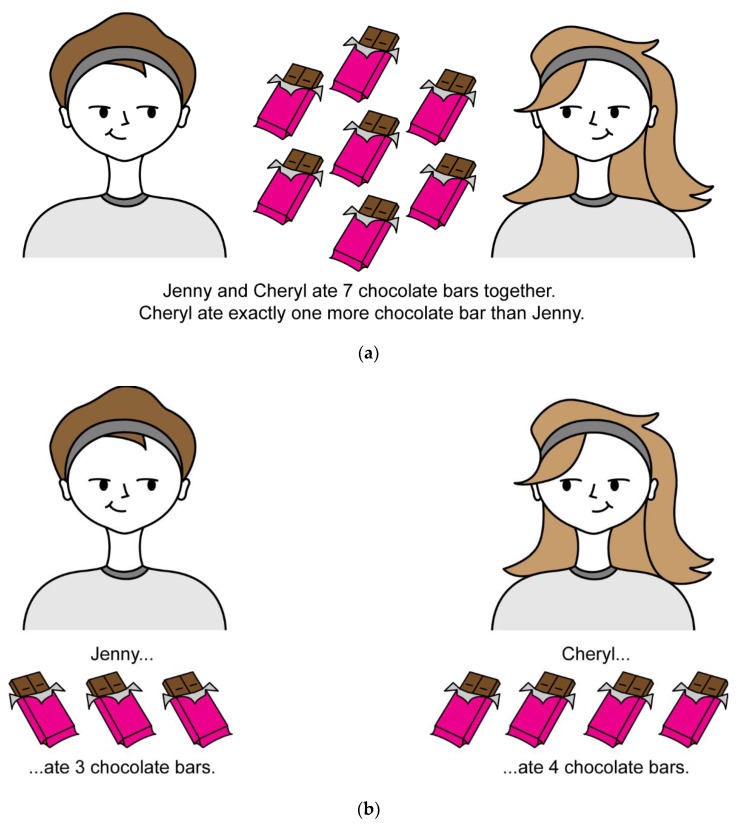
(**a**) Indirect statements are given about the relationship between Jenny, Cheryl, and the number of chocolate bars eaten by each. Logical inference is required to deduce what the information is, that Cheryl ate four chocolate bars, while Jenny ate three. Accurate representation of the world in this situation depends on extra processes required to logically infer the information about the world. (**b**) Direct statements describe clearly the relationship between Jenny, Cheryl, and the chocolate bars. There is no inference required as the statements give the information directly: Cheryl ate four chocolate bars, and Jenny ate three chocolate bars. Accurate representation of the world in this situation relies on vivid representing, where the information about the world is conveyed directly without need for inference.

One approach is making knowledge vivid. Vividness is defined by Levesque [2] as a knowledge base (KB) having two main properties (see Figure 2). First, a one-to-one correspondence should exist between symbols in the KB and objects of interest in the world. Second, a connection between symbols in the KB exists if and only if a relationship also exists among objects in the world.

**Figure 3:**
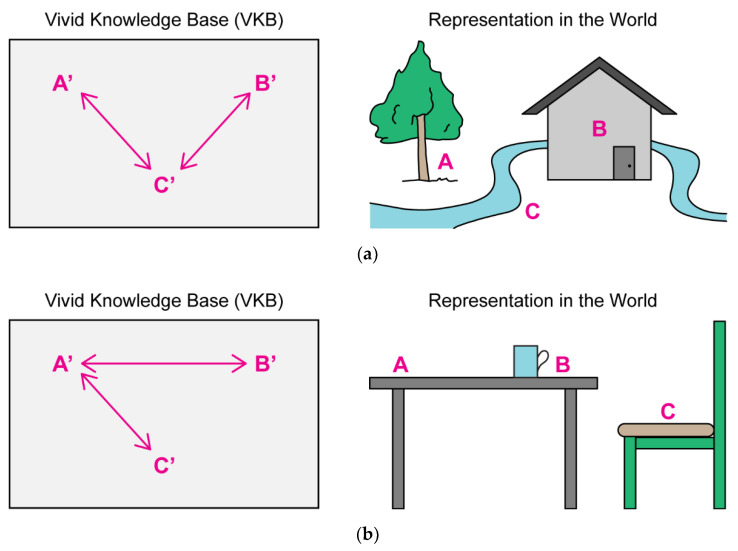
The computational definition of vividness from Brachman and Levesque [7] highlights the assumptions necessary for a KB to be considered vivid. It is understood that the KB assessing a world α operates under the “close-world assumption”—if something is not known to be true or to exist, it can be considered false or non-existent. Thus, a VKB will have as many representations as there are elements observed in a world α, no more, no less. For the elements that do exist in a world α, the KB will have representations with a one-to-one correspondence to each of those elements. If there exist any relationships between the elements in the world α that can be observed, the corresponding representations for those elements in the KB will also be connected. Any changes to the elements present in a world α will also be immediately reflected in the KB. (**a**) In the world, there exists a tree (A), a house (B), and a river (C). Both the tree and the house stand next to the river, but the house does not stand next to the tree. In the vivid knowledge base, there is a one-to-one representation of each of the elements found in the world (A’ is the representation of A from the real world, B’ represents B, C’ represents C). There are no more representations than there are elements to represent in the world (there exists no D’ in the VKB if there is no element D observed in the world). The relationship between each representation in the VKB reflects the relationship between the respective elements they represent in the world. For example, as the tree (A) stands next to the river (C), that relationship is also represented between A’ and C’ in the VKB. Conversely, since the tree (A) and the house (B) are not standing next to each other, there is also no relationship between A’ and B’ in the VKB. (**b**) In the world, there exists a table (A), a mug (B), and a chair (C). The mug stands on the table, and the chair is next to the table. In the vivid knowledge base, there is a one-to-one representation of each of the elements found in the world (A’ is the representation of A from the real world, B’ represents B, C’ represents C). There are no more representations than there are elements to represent in the world (there exists no D’ in the VKB if there is no element D observed in the world). The relationship between each representation in the VKB reflects the relationship between the respective elements they represent in the real world. The relationship between each representation in the VKB reflects the relationship between the respective elements they represent in the world. For example, as mug (B) sits on the table (A), that relationship is also represented between A’ and B’ in the VKB. Conversely, since the mug (B) is not standing on the chair (C), there is also no relationship between B’ and C’ in the VKB. Note that if there is a floor that the chair and table are standing on, it is not shown or observed by the KB, and thus it cannot be represented in it.

The knowledge should also be complete, such that we know which statements are actually true. Consequently, this type of KB presents a model of the world it describes, since any type of information about the world can be extracted from the KB (see Figure 3). For example, to know a certain fact about Jenny, we would look through the KB for propositions starting with “Jenny is a…”, similarly to a search through a database. Accordingly, a change in the KB would correspond to a change in the world [2,6].

Since a lot of the information in a proposition is redundant, the KB’s form can be optimized into a database or semantic network. This will enable a very large amount of information to be handled at the same time allowing a holistic approach to reasoning. Following this reasoning, Levesque [2] suggested that human reasoning may be governed by symbol manipulations of vivid knowledge representations and nothing more. 

This view is appealing as vivid information is processed by people all the time. It exists in maps and pictures, and it provides us with concrete and explicit answers to questions. It also allows a weaker type of logic which deals with disjunctions. Not all types of information can be considered vivid, such as linguistic information. However, they can be forced to be [8].

### 2.2. Limitations

Problems with vividness as presented by Levesque arise especially from the assumption of a closed world, which deprives the database from the possibility for uncertainty, or for changes to the information stored over time. Additionally, some inferences are difficult to make without the possibility for incomplete knowledge, such as whether the unstated is an indicator of true absence, or an indicator of the lack of knowledge on the presence of something [9].

Kautz, Kearns, and Selman [10] elaborated Levesque’s model and proposed the use of a characteristic model for a Horn theory. A Horn theory is a set of disjunctive propositions with at most one positive proposition. A characteristic model provides the minimal set which entails all other elements of a Horn theory in it. In such a set the intersection of any two elements will compose any other element. This allows a relaxation of the completeness limitation, since an intersection of numerous characteristic models can encompass all the information in its set. Furthermore, Kautz et. al. [10] were able to show that sets of models were as efficient as logical formulas, and sometimes even gained substantial savings. However, Gogic, Papadimitriou, and Sideri [11] pointed out the difficulty of implementing this idea. They pointed out that finding the Horn formula which has the biggest set of models is an NP-hard problem. 

Etherington [8] claimed that vividness is the basis for commonsense knowledge. The author suggested an architecture which relies on a vivid KB but is overall supported by a more general reasoning such as first-order logic. Some of the information that enters the system can be “vivified” and stored in a vivid KB (VKB). However, some information is incomplete and must be stored separately. Consequently, a hybrid model was suggested. In this model a query is answered by referring to the VKB. If the query cannot be adequately answered, the system delegates to the original information and some other type of reasoning is used to answer the query. Ultimately, the usefulness of the model will partially depend on the proportion of queries answered using the VKB. 

### 2.3. Reasoning beyond Vividness

Although VKBs provide complete and tractable knowledge, they are restricted in representing knowledge. Brachman and Levesque [7] considered some potential extensions to VKBs that preserve tractability.

First, they discussed the notion of incomplete sets of literals, such that the closed world assumption does not apply. Considering a small set of logical formulas, α, Brachman and Levesque proposed to transform α into conjunctive normal form (CNF). Such a conjunction consists of more conjuncts that are composed of disjunctions of one or more literals. Consequently, a set of clauses c1, c2, …, cn, and all tautologous clauses that contain an atom and its negation are discarded. The property that follows is that a KB entails α if and only if the KB entails c*i* for every remaining c*i*. These conditions provide tractable reasoning in the absence of complete knowledge. This, however, is only useful for a prepositional language; the authors were not clear on how this notion can be extended to logic with quantifiers. 

Next, they proposed the incorporation of definitions. Suppose α is a formula which uses predicates in the KB, and p is a predicate that is not a part of the KB. A sentence can be created that serves to define new predicates using old ones; it can even be recursive. Reasoning with such a vivid KB remains efficient. A query that contains p, a newly defined predicate, can be replaced with α. 

Finally, the authors considered a type of hybrid reasoning which incorporates various forms of special-purpose reasoning algorithms. Such a system should use procedures such as equation solvers as well as first-order reasoning. Semantic attachment is considered, in a sense that procedures are associated with a particular function and predicate symbols. These expressions are simplified, if possible, before passing them to a specific resolution algorithm. A more general version of this notion is called theory resolution. This theory is built into the unification process itself. The special-purpose reasoner will then resolve clauses which have complementary literals.

The theoretical constructs and limitations of vividness presented thus far by Levesque [2,7] and subsequent contributors are consistent with the symbolic approach to vividness. The discussions around vividness in artificial intelligence (AI) are still very recent. However, vividness linked to semantic memory, imagery, and mental models has had a long history in cognitive psychology, with new emerging links to cognitive neuroscience. Knowing that systematic and non-symbolic approaches to vividness in AI, such as neural networks, take inspiration from current understanding of human cognition, it is important to review the contributions stemming from the philosophical and neuroscientific fields to the current understanding of vividness. 

## 3. Vividness in Natural Cognition

### 3.1. Vividness in Philosophy and Phenomenology

Historically, vividness has been a key phenomenological aspect related to the nature of human condition and communication in several traditions of philosophy [12], psychology (see the pioneers such as Galton [13] and Betts [14]), and neuroscience [15]. One of the earliest mentions of vividness (ζωντάνια) appears in Aristotle’s analysis on the power of tragedy in evoking “vividness of impressions in reading as well as in representation” [13], including the corollary distinction between vividness related to reading (i.e., indirect, conceptual) and vividness related to impressions from perception (direct, sensory). Two thousand years later, David Hume described the immediate ideas or images that (within networks of associations) exert a prepotent influence on imagination through poetry and fiction: “The vividness of the first conception diffuses itself along the relations, and is conveyed, as by so many pipes or canals, to every idea that has any communication with the primary one” [16]. Closer to our days, elaborating further on Brentano’s approach [17], Meinong reworked Aristotle’s and Hume’s distinction between ideas and impressions (fantasy presentations vs. perceptual presentations [16]) as intensive gradient of vividness (*Lebhaftigkeit*, [16]), not dependent on content of the underlying representation, but on availability to consciousness in the form of discrete temporary episodes of awareness [18]. 

Arguably, both Brentano and Meinong used vividness intuitively as a natural kind, a primitive notion, and such a perspective was influential and bore fruitful empirical contributions in relation to a particular brand of experimental phenomenology in the early 1900s known as the Graz School of Gestalt Psychology [17]. Experimental phenomenology is “the study of appearances in subjective awareness” [19], and it refers to “both a theory and a method derived from, but not identical to, Gestalt psychology” [20]. A concept similar to the current notion of vividness—*phenomenal salience* (*Auffälligkeit*)—was central in the experimental phenomenology of Vittorio Benussi [21,22], and of Bonaventura [23] and Calabresi [24], who essentially applied Benussi’s ideas in the more specific area of the study of the conscious awareness of the present (see [20,25,26,27,28]), albeit with different terminology (notably, Benussi’s salience was at times dubbed as “vivacity”, a synonym for vividness, see [17]). 

In the context of perception (during the so-called Graz period) and in line with Brentano’s and Meinong’s frameworks, Benussi conceived of salience as a *clustering* process, driving the distribution and direction of attention and shaping the *qualitative* aspects of experience as directly given in awareness, specifically, as “presentation” (*Vorstellung*) [29]. Crucially, salience as a property followed the same relational functional feedback logic of productions [30,31]. Salience was seen as a mechanism which automatically and unconsciously could leverage founding properties (so-called *inferiora*) triggered by real underlying object/reality such as low-level sensory processing and selective attention, which had direct stimulus-bearing causal efficiency on the body, as the constituting structure of emergent perception and conscious experience. The latter complexes in turn could be intentionally put in the foreground of awareness, as non-sensory presentations, and be manipulated at the higher, relatively more complex levels (so-called *superiora*) of thought and imagination and other functional relational activities. Via this sort of amplification, operated by attention, and driven by salience, the latter types of higher-level presentations could feedback loop to the *inferiora* level, and depending on the conditions, then generate, to different extents of intensity, the effects (or outputs) of actual sensory stimulation. (Indeed, Benussi was a pioneer in demonstrating experimentally that even when some of the structural elements that constitute illusory phenomena, e.g., the Müller-Lyer illusion, are not given through direct perception, but only represented in imagery, they could occur with an almost equal intensity as optical visual illusions [32]). 

Thus, for Benussi salience was a specific characteristic of perceptual and time consciousness [26,33], which worked at a microlevel of the present conscious awareness. However, in the latest phase of his work (during the so-called Padua period) Benussi [34] refined further the concept of “non-sensory presentations” as “representations” (*mental presentations*) of absent properties (unrealities) which extended the range of several other complex “intellectual” mental acts beyond those previously studied in the Graz period: these included verbal semantic memory, dreaming, imagination, imagery, suggestion, and hallucination.

Based on an interpretation of, and integration with, Benussi’s salience, it is therefore possible and desirable to attempt a more explicit definition of the current concept of vividness grounded from within experimental phenomenology, since, surprisingly, none of the available phenomenologically-inspired definitions is neither clear nor satisfactory, namely, none goes beyond a vague appeal to a self-evident primitive and intuitive notion which stands on equally ill-defined synonyms (for review see [17]). 

When remembering specific everyday objects or events linked to past experiences, such as personal events (e.g., the face of a relative or a pet), people generally report “seeing with the mind’s eye” [35]. A pervasive aspect of people’s report is the vividness of their mental images [13]. Images may come from the imagination (e.g., a pink dog) or from retrieved episodic and specific representations which refer to everyday objects (e.g., your breakfast this morning). Thus, we can define vividness as:

*(i)*  *The extent to which mental images reflect the composite (or cluster) qualities or qualia (including specificity, detail, and richness) of unreal* [34] *visual representations that would have been generated if the object had actually been perceived (or real, Benussi* [34]*).*

Let us consider some key aspects of this definition.

First, (i) we need endorse no presumptions about the underlying format of mental images (e.g., propositional or pictorial) other than that they are a type of analogue. All that one needs to assume is some elementary properties of databases. That is, a memory database containing information about a given domain (of objects and relationships between these objects in the world) will contain individual images that consistently designate individual objects in the world and relationships between individual objects that designate the respective relationships in the world [7]. Principle (i) is in total agreement with the fundamental premise of experimental phenomenology, that all mental representations and activities are about something and about objects (see “*premessa a*”, in Benussi [34] and Benussi [28]). The key ingredient in the latter statement is Brentano’s notion of intentionality (“reference to something as object of consciousness” [29]) which as we will discuss later is critical to properly frame the notions of representation and information.

Second, according to a possible interpretation of (i) (which is compatible with but not identical to the Brentanian or Benussi’s views), vividness may be conceived as a crude proxy for what is available in the memory database; a report about a represented object X or relationship involving X will be more or less vivid depending on the intensive gradient to which information about X is perceived to be complete or detailed, and in turn this should be reflected in behaviour, e.g.,, the time needed to respond to a query about X. (The latter connects directly with classic verificationism in psychology [36]). Indeed, similarly to vividness as sketched above, in emphasizing that salience is a self-evident, primitive, and intuitive concept, Benussi maintained that “we recognize it with confidence and we attribute different degrees of intensity to it: there are different degrees of salience just as there are of diversity, weights, for wrath and joy. The mental experience that is defined as “experience of salience” (*Auffälligkeitserlebnis*) must also be able to change in intensity, because at some point it ceases; there are, states of maximum, medium and minimum “consciousness of salience” (*Auffälligkeitsbewusstsein*)” [27].

An initial comment is in order on what assumptions are introduced when speaking about qualia of visual representations. Visual representations, perceived or imagined, can be studied by analyzing single relevant psychological factors. The simplest view is that vividness is one attribute that expresses (i.e., makes explicit) the combination of these factors to consciousness, and by virtue of this many-to-one function, variations in the attribute of vividness determine in a probabilistic fashion measurable processing differences in consciousness. The statement that vividness can apply to both perception and imagery has already received some empirical support in experiments which have compared vividness judgments on perceived and imagined visual patterns [37,38,39]. 

The previous definition implies that vividness should be measured comparatively between the perceived quality of the visual mental image and the actual seeing [40,41]. Perceived image quality can be operationalized in several ways, including rating, latency, priming, and recall. By anchoring one end of the vividness scale to the vividness of a percept, this definition provides an objective foundation for what is still a subjective judgement, similar to the principle used in classical psychometric scaling [42]. The extent to which the various operationalizations agree then provides evidence for the unity and usefulness of the concept of vividness, despite its inherent subjectivity. This approach makes vividness a construct that permits to interface first-person and third-person psychological functions, therefore, central to consciousness [43,44]. 

Finally, the proposed characterization has some key methodological implications for communication and meaning. Vividness, as discussed for example by Perky [45], is prevalent in laypeople folk’s conceptualization of mental imagery and is entrenched, for instance, in language we use to describe dreams and episodic memories, all uses that are coded in the major dictionaries. At least since the British empiricists headed by David Hume [46] and then Vico’s new science philosophical system [47], it has been long accepted and implied in several Indo-European languages that mental images are typically less vivid than real percepts, which comparison involves an implicit scaling of the one to the other. Consequently, communication about vividness between the experimenter and the subjects can be carried out in a relatively straightforward manner with levels of ambiguity as tolerable as other psychological constructs defined by reference, since one does not really need to explain the meaning of vividness through many or long sentences or words, definitions, or adjectives to elicit subjects’ understanding. Reference to seeing and dreaming is usually enough to explain ostensibly imagery and vividness to most people (except 2–3% of the population who report no experiences of visual images, or the so-called aphantasia, see later); as early as 3 years of age, children can understand what the request to give verbal report on imagery entails [48].

According to Benussi [33], it is precisely by virtue of the mediating function of words to “conduct to” the vividness of meaning of the referred objects (“…la funzione psichica viva del capire, del comprendere…[the vivacious psychic function of understanding, of comprehension]” [33], that unreal absent objects which are not perceived as being before us, as present, can be reinstated as having causal efficiency and being present to consciousness as real currently perceived objects (again, following the causal feedback logic of productions within the dynamic interaction between *inferiora* and *superiora*). It could be noted that Benussi’s account is compatible with the account of dynamic transfer or selection of imagery information from long-term memory (LTM) into working memory [49]. However, Benussi’s account contributes a bit much more explicitly to a hypothesis on what that dynamic mechanism of information processing might feel like in our consciousness. That is, phenomenologically, past, or even prospective, memories may become vivid when shifted to the focus of our present consciousness. Accordingly, perhaps the most important added contribution of the proposed definition is that the central epistemological issue regarding the nature of “representation” and its underlying “information” can be somehow resolved by a pragmatic integration of experimental phenomenology and information processing traditions. The properties of the data base as discussed following Benussi’s definition are not “elementary” in the classical sense, as for example in the sense of a bit of information per time unit. Elementary properties according to Benussi are qualitative in nature (as opposed, for example, to Levesque’s properties of data base). Similarly, the meaning of (mental) presentation, in fact, is different from the more usual “Representation” as it will be reviewed for AI, cognitive science, and neuroscience, where it refers to the computational elaboration of physical stimuli by the mind/brain. The information contained in mental presentations is “qualitative in nature, endowed with meaning, and not merely a product of the computational representation, retrieval, and elaboration of physical stimuli” [20]. Phenomenological information in other words is not just reducible to quantitative capacity of transmission and elaboration of a signal as in information theory [50]. However, these two different constructs are coalescent, since information capacity and elaboration in the physical substrate of brain activity correlates with context-bound referential communication which is embedded in mental activity [51]; consequently, in this view, representation and information not only reflect the intensity of the message/experience but also reveal features of what the message content is about, or not, plus its implied meaning and functional or adaptive role [52]. It is this sort of embedded intentionality which gels both qualitative and quantitative aspects of un/consciousness (and vividness).

The issue of vividness of perceptual experience is having a comeback and is now a hot topic in the perceptual and cognitive quarters of psychology and neuroscience. There have been numerous names and labels assigned to the concept over the years and the construct has had a conceptual evolution/development in many different directions and sub-fields (see discussion in [1]). The bulk of research can be found in the traditional psychological construct of vividness, specifically of perceptual or memory (often called “mental”) imagery [40,41]. 

Haustein, Vellino, and D’Angiulli [1] performed the complete bibliometric analysis of the peer-reviewed literature on vividness between 1900 and 2019 indexed by the Web of Science and compared it with the same analysis of publications on consciousness and mental imagery. While they observed a similarity between the citation growth rates for publications about each of these three subjects, an in-depth analysis shows that these concepts rarely overlap (co-occur) in the literature, revealing a surprising paucity of integration among these concepts taken together. A disciplinary analysis shows that psychology dominates the topic of vividness, even though the total number of publications containing the latter term is small and the concept also occurs in several other disciplines such as computer science and artificial intelligence. The findings suggest that without a coherent unitary framework for the use of vividness construct in research, important opportunities for advancing the three fields (including consciousness and imagery) might be missed. (Indeed, the present chaotic situation has led some philosophers to call for the abandonment of the concept of vividness altogether, see [49]). To counteract this nihilist position, Haustein et al. [1] suggested that an evidence-based framework will help to guide research from all disciplines that are concerned with vividness and help to resolve the challenge of integration.

Motivated by the integration challenge, we attempt to flesh out, empirically, some of the “many pipes and canals” mentioned by Hume. Specifically, we investigate the network of relationships linking phenomenology of vividness, implicit perception of the temporal present in visual mental imagery, and brain activity. To such end, in the following sections not only do we attempt to integrate different theoretical traditions, but also we integrate methodological practices from experimental phenomenology, human symbolic information processing, and distributed parallel processing [53].

### 3.2. Vividness in Psychology

#### 3.2.1. Vividness as a General Function of Perceptual Consciousness and Action (Marks’ VVIQ-Vividness)

**Figure 4:**
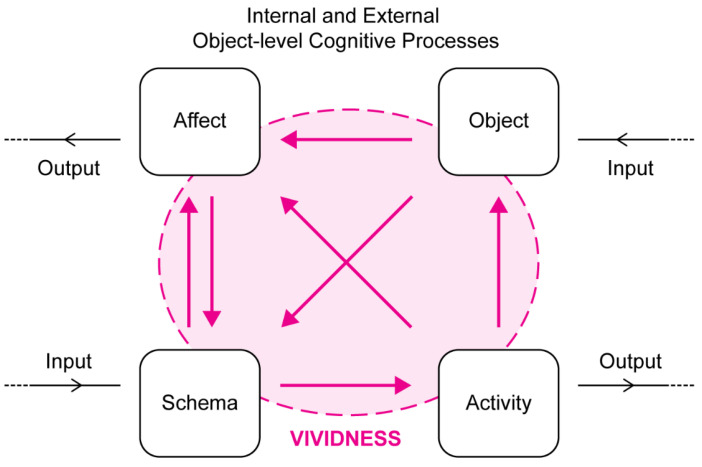
According to Marks [56], four key processes guide the conscious assessment of the surroundings—affect, schema, object, and activity. The cycle of activity triggers the formation of mental images of varying levels of vividness to help with the assessment of an observed external activity or with an internal goal. It is the degree of neuronal activation between those four key processes that determines the level of vividness of the perceived mental image, as well as the vividness of the sensations associated with it. Figure adapted from Marks [56].

Without question the most influential view of vividness in psychology is linked with David Marks’ General Theory of Behaviour [48] and the Vividness of Visual Imagery Questionnaire or VVIQ [54]. Marks’ General Theory [55,56] is founded on the assumption that the primary motivation of all of consciousness and intentional behavior is psychological homeostasis. Psychological homeostasis is as important to the organization of mind and behavior as physiological homeostasis is to the organization of bodily systems. Consciousness processes quasi-perceptual images independently of the input to the retina and sensorium. Therefore, central to this theory is the construct of imagery vividness, defined as the combination of clarity and liveliness, which is instrumental to all forms of consciousness: imagining, remembering, thinking, feeling, predicting, planning, pretending, dreaming, and acting. Accordingly, “a mental image is a quasi-perceptual experience that includes action schemata, affect, and a goal (see Figure 4). Conscious mental imagery serves a basic adaptive function in enabling a person to prepare, rehearse, and perfect his or her actions. Mental imagery provides the necessary means to guide experimentally and transform experience by running activity cycles as mental simulations of the real thing. Such activity rehearsal can only proceed effectively when the rehearsal incorporates vivid imagery. Imagery that is vivid, through virtue of being as clear and as lively as possible, closely approximates actual perceptual-motor activity, and is of benefit to action preparation, simulation, and rehearsal.” [56].

One of the ways in which this phenomenological construct has been empirically investigated and validated has been through introspective reports and the VVIQ [57] and converging objective indicators, especially neuroimaging correlates [58]. A significant body of work shows that vividness of visual imagery is determined by the similarity of neural responses in imagery to those occurring in perception of actual objects and performance of activities. Consciousness is the control, executive center for integration and regulation of thoughts, feelings, and actions with vividness and conscious mental imagery as foundation stones. It is interesting that in Marks’ account vividness does not refer to images as “pictures in the head” but rather to various forms of activity or action in a complex cycle which provide the toolbox for the individual to reach desired psychological homeostasis.

#### 3.2.2. Vividness as Episodic Memory

When remembering specific everyday objects or events linked to past experiences, such as personal events (e.g., the face of a relative or a pet), people generally report “seeing with the mind’s eye”. A pervasive aspect of people’s report is the vividness of their mental images. Images may come from the imagination (e.g., a pink dog) or from retrieved episodic and specific representations which refer to everyday objects (e.g., your breakfast this morning). Setting aside “imagination imagery”, whose vividness is entirely subjective, we can define the vividness of “realistic” imagery as the extent to which mental images reflect the composite quality of visual representations that would have been generated if the object had been perceived.

In such case, the memory of these internal images in an individual is considered to be a database of inner representations. It would contain information about a given domain (of objects and relationships between these objects in the world), and this domain would in turn contain individual images that consistently designate individual objects in the world and relationships between individual objects that designate the respective relationships in the world. Since the recall of these internal mental images is highly dependent on long-term memory retrieval, it can also be said that vividness of images is also highly dependent on memory, since the composite quality of the perceived images fades as retrieval of its specific details becomes difficult. For example, mental imagery also plays a role in self-perception and mental time travel [59]. Vivid mental imagery is correlated with a heightened, more acute sensory perception of the world. Individuals with vivid imagery will experience memories of the past in greater details. Similarly, they will also perceive their mental projections to the future in greater detail, as both abilities to project oneself in the past and future are correlated [59].

However, vividness is not just a matter of detailed mental imagery. As Thakral et al. [60] suggested, regions of the brain are differentially responsible for the level of detail and vividness of a mental projection in the future, or episodic simulation. Whereas the brain’s core network is associated with episodic simulation as a whole, regions such as the hippocampus are correlated with vividness of visual projections (i.e., how close to reality the visual projections appear to be), while other regions such as the lateral parietal cortex tend to respond to the level of episodic details in the episodic simulation.

Furthermore, retrieval of vivid mental images depends on memory, and memory can be distorted and altered through any misinformation that is fed to an individual [61]. As previously pointed out in a review by Gilboa [62], a fine distinction between autobiographical memory retrieval and episodic memory retrieval also exists. This distinction can be used to help differentiate a true memory from a fabricated or altered memory. At the neurological level, while both types of memories engage the prefrontal cortex, episodic memory retrieval is distinguished by the activating the right mid-dorsal prefrontal cortex, which is not active for autobiographical retrieval. Conversely, the regions of the left ventromedial prefrontal cortex are mainly active during autobiographical memory retrieval, and not in episodic memory retrieval [62]. Such differences allowed to identify that episodic memory involves, at the psychological level, conscious and deliberate monitoring of information during recall. In contrast, autobiographical memory recall is quick, without conscious interference and relies on a sense of ‘knowing’ [62]. This shows that vivid mental images can be either/both retrieved from the memory database or/and they can be consciously manipulated or created.

#### 3.2.3. Vividness in Cognitive Science

Pylyshyn [63] discussed mental imagery, visual perception, and the intersection between the two. According to him, accessing information from a visual scene is different from examining a mental image. A major difference lies in the fact that mental images represent the conceptual content of a scene. This enables mental images to be distorted throughout time in defined, conceptual ways. Unlike a torn photograph, a mental image can miss a discrete object, could misplace an object, or fail to link its various properties. 

This characteristic of mental images also prevents them from being visually determinate. A picture, in comparison, will always have a defined size, shape, spatial relationships, and many other characteristics, that could be left unspecified in an image. Thus, images are not only iconic but also extensively interpreted. 

Pylyshyn [63] discussed why favoring a picture theory of mental imagery is problematic. Since a person can decide on the content and properties of a mental image, this suggests a choice; one must have a conceptualized content, an interpretation, a description, or all three in mind. For example, when constructing a shape in one’s mind, she cannot mistake it for any other, as she might with a real figure. (It must be noted that most of Pylyshyn arguments update identical or similar points first raised in Sartre’s philosophical and phenomenological analysis [12]).

The main caveat to these claims is that to “mentally image” something corresponds to imagine seeing something. As a result, some aspects of the image must be assigned a value. For example, when imagining Jack and Kate beside each other, one would be inclined to imagine Kate to the right, or left, of Jack. Other aspects are not mandatory such as the type of clothes they are wearing. 

Another problem is that from an informational point of view, no display is identical to a mental image. No visual stimulus entails an interpretation of a picture. Mental images do and are accordingly conceptual. It is possible that they just might be nothing but an interpretation of previously processed information.

There is no doubt that generating a mental image has a connection to seeing a visual display. The main assumption that Pylyshyn [63] questioned is that generating a mental image consists of interpreting a special form of information which is picture-like or “depictive”. The latter suggests that the image is pre-conceptual or pre-perceptual and therefore subject to interpretation by an early stage of vision. 

This leads to the question of what is meant by the term “depictive”. Pylyshyn [63] claimed that it is not enough to have an accurate representation of geometrical, spatial, and visual properties; this would suggest that linguistic representation is depictive. He then explored the importance of semantics in trying to decide which side rules the debate of the nature of images. Questions such as “Does being depictive require a spatial form of representation?” and “Does being spatial require that images preserve metrical spatial information?” were posed. The conclusion: neuropsychological evidence is incapable of resolving the debate. 

Overall, Pylyshyn [63] hypothesized that the form of representation present in vision is similar to visual imagery. Plausibly, the content encoded in both has a propositional form. Another question discussed was whether there is anything special about representation underlying visual imagery. As we will review shortly, many argue that is the case [64], but such a claim must be empirically sustained. So far, no theory has been able to account for all the findings concerning mental imagery. Thus, throughout the search for a comprehensive unfied theory it is important to consider some constraints. 

Throughout extensive research we have come to know the semantic and syntactic properties of formal logic as ways of encoding knowledge. It should therefore serve as a baseline and a constraint on a theory of imagery. Additional related constraints on representations underlying imagery are also relevant in this context. 

Similarly to Levesque’s [2] vivid KBs, Pylyshyn [63] considered properties of image representations such as a lack of explicit quantifiers, disjunctions, and negations. Other properties include information about relative locations, which represent discrete individuals and physical as well as abstract properties. Moreover, the information about objects in a mental image can be associated with objects in a visual scene. Indexes may be used to bind imagined objects to objects in a scene. This notion is similar to the one-to-one correspondence between objects in the world and in the KB, proposed by Levesque [2].

Pylyshyn [63] noted that there are no theoretical ideas that consider any of the restrictions discussed, Levesque [2] being the exception. He postulated that these special characteristics of visual representations are restricted in some of the same ways that vision is, and ought to be a step towards a unified theory of mental imagery. 

#### 3.2.4. Vividness, Simulation and Mental Models in Human Reasoning

Goebel [64] proposed that the best way to pin down the difference between depictive and linguistic, direct and indirect, representation is within a computational context. Observing the effectiveness of problem solving via the two representations can provide insight on the computational methods applied. 

Initially, it appears that depictive representation is much easier than linguistic representation. For example (adapted from Goebel [64]), left-of (A, B) can be interpreted with means of first-order semantics. Contrarily, the interpretation of A B seems to be available by inspection and occur automatically as part of our visual perception. Goebel suggested that depictive perception might result from rapid retrieval of some highly compiled form of visual reasoning. However, it is still far from obvious which representation is better, or more efficient, and less computationally costly.

Levesque [2] did not distinguish information into depictive and linguistics representation per se. He simply claimed that vividness makes computation much more efficient as it captures a vast amount of data as false (anything that is not stated in the KB). This suggests that different representations, such as visual, spatial, and linguistic representations differ in their ability to capture knowledge. 

Logic also plays an important role in any form of representation. One can decide the soundness of reasoning independent of the form. For example, inspection can relate soundness to accuracy. If any form of representation does not reflect reality in an accurate way, it will lead to an erroneous conclusion. Similarly, the concept of completeness and consistency (p and ~p is impossible) can be determined regardless of form. 

Goebel [64] illustrated the importance of logical concepts in cases where depictive representation leads us down the garden path. For example, the Mueller-Lyer optical illusion of apparently unequal lines fools our visual perception when we do not follow logical principles. In his view, any form of representation should adhere to principles of soundness, completeness, and consistency. 

#### 3.2.5. Vividness in Neuroscience and Neuropsychology

**Figure 5:**
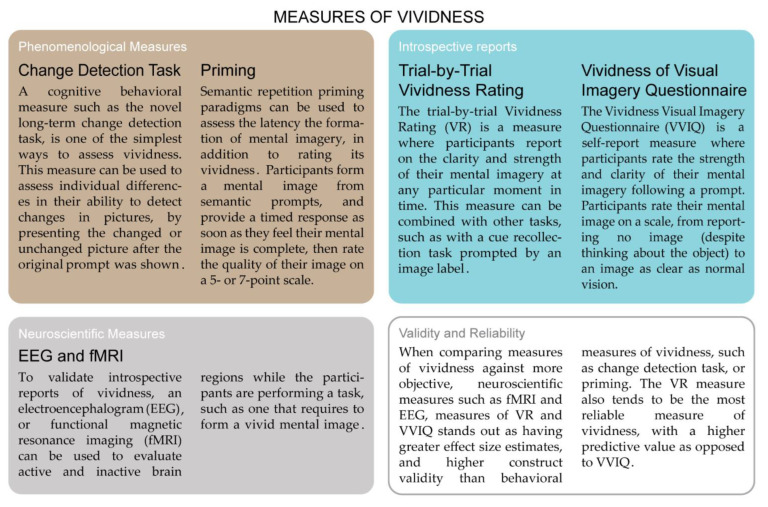
An overview of past and current measures of vividness in cognitive sciences. These measures are generally used to assess individual differences in mental imagery vividness. Phenomenological Measures: change detection task [65], priming [17]; Introspective Reports: trial-by trial vividness rating: [66], vividness of visual imagery questionnaire [55]; Neuroscientific Measures [67,68]; Validity and Reliability [69].

Neuroimaging studies have been established as a good technique for inferring the nature of visual imagery but over and above to provide objective correlates of subjective self-reports about vividness (see Figure 5). Patterns of neural activation can be compared during imagery and perception of same visual content. After establishing a baseline, activation during mental imagery can be further investigated to gain insight into the vividness of the mental image. Two types of objective neural correlates of vividness have been confirmed, direct objective neural indicators of within-subject gradients of vividness (variations of intensity of self-reported vividness at individual level or repeated measures) and behavioral/neuropsychology correlates. An exhaustive meta-analysis has shown clearly that direct neural indicators yield significantly higher predictive associations than behavioral measures [47].

Research suggests that the underlying neural activity of mental imagery and visual perception is similar [70]. Amedi et al. [71] investigated these patterns of brain activity in visual object recognition (VO) and vivid mental imaging (VI). Using the fMRI BOLD signal, some fundamental differences emerged. A negative activation was observed in the bilateral auditory regions in response to VI. Contrarily, a baseline activity was observed in response to VO. Deactivation was also observed across later auditory areas in VI, corresponding to activation in VO. Overall, the absolute magnitude of activation was higher for VI and consistent across all participants. 

Furthermore, Amedi et al. [71] investigated the effect of vividness on brain activation. A significant positive correlation was established between image vividness and magnitude of deactivation in the primary auditory cortex (A1) and somatosensory cortex. A positive correlation was also established between vividness and activation in visual areas. They postulated that deactivation of non-visual areas and activation of visual areas aid in creating a more vivid mental image. Presumably, a more vivid image is less affected by non-visual sensory modalities and bottom-up processing. They further claimed that differences in activation between VO and VI are critical to create the experiential difference between perception and imagery. 

Gonsalves et al. [67] showed a different pattern of activation which was correlated with the gradient of variation of vividness within and between subjects. This occipital brain potential labeled P850 was also shown to be higher in response to visual mental imagery, specifically in retrieval from memory. Bodily response also plays a role in the measure of imagery vividness associated with emotional experiences. Vianna et al. [72] proposed that in highly vivid imagery experience, the central nervous system has a greater effect on emotional experience. In low vivid imagery experience, however, bodily information from the gastrointestinal system plays a more significant role.

**Figure 6:**
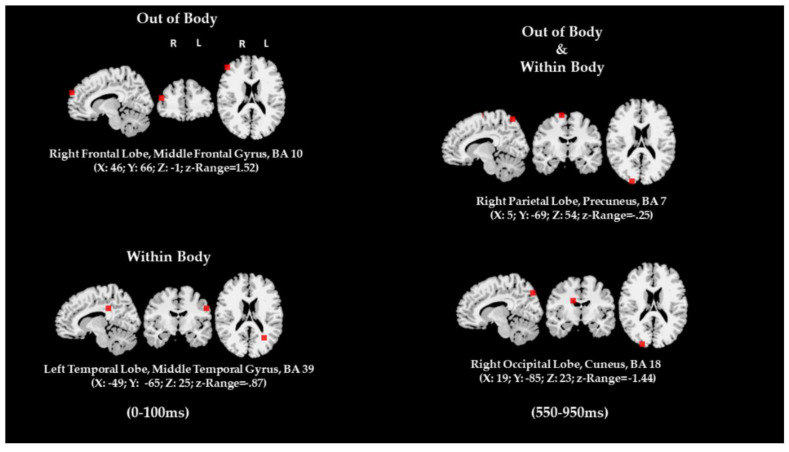
Vivid imagery experienced outside and within the body shows both similar and distinct loci of activation in the brain. Figure taken from D’Angiulli et al. [77]. Red points indicate the estimated anatomical graphic location of the generating source of EEG activity on the structural MRI template slices (i.e., sagittal, coronal, axial); the formal label, Talairach coordinates, and millisecond detection time intervals are given below the template according to Broadman Areas (BA).

Another measure of vividness can be established by comparing high-vivid individuals with low-vivid individuals. Some clear differences in patterns of activation emerge. The abovementioned ERP signature, P850, has been shown to differentiate between individuals with high-vividness vs. low-vividness imagery. The best characterization of this ERP signature has been provided in several studies by Farah and colleagues [70,73,74,75]. Specifically, Farah and Perronet [73] reported a stronger P8/900 in the occipital electrodes for participants who reported more vivid images. The actual millisecond range of the waveform has been reported as positive voltage maximally peaking anywhere between 800 and 1100 ms. The localization of the neural generators of the LPP signals was initially estimated to correspond to two network dynamics, one going from the medial to lateral occipital electrodes, and a second one going from the medial occipito-temporal to lateral fronto-temporal electrodes [74]. A follow up study measuring functional magnetic resonance Iimaging (fMRI) activity [76] confirmed the visual association cortex was engaged during the mental image generation. The left inferior temporal lobe was activated across participants, some of whom showed activity of the occipital association cortex. Similar objective correlates have been found by correlating VVIQ scores and fMRI BOLD activation [58,68]. Most recently, these findings have been firmly confirmed (see Figure 6).

Furthermore, it has been found that people who are better at visualizing exhibit interaction between brain areas that code for color perception and color naming [68]. Others examined vividness across sensory modalities, namely, visual, gustatory, kinesthetic, tactile, and somatic [78]. The latter study found that activation in areas corresponding to modality-specific perception also occurs in the process of imagery for those modalities. This was especially evident in high-vivid individuals. Consequently, the ability to generate a vivid image depends on creating representations which correspond to neural mechanisms of perception. A study by Pearson et al. [79] showed that vividness was associated with the likelihood in which an imagined pattern would appear dominant when the participant was subsequently presented with a binocular rivalry display which contrasted the pattern with a similar distractor. Self-report questionnaires measuring imagery vividness also predicted individual differences in the strength of imagery effect.

Additionally, Imms et al. [80] demonstrated the correlation between the number of neural connections firing at once during processing and information processing speed. In the context of vividness, this hints at high-vivid individuals benefitting from a richer connectivity in their neural networks and processing speed.

It should be noted that some individuals may not be experiencing visual imagery vividness. Zeman [81] investigated individuals that lack any inner visual representation, while being otherwise healthy. This absence of a mind’s eye is referred to as ‘aphantasia’—the absence of mental visual representations—and is estimated to be affecting around 2–3% of the population [81]. In their wakeful state, individuals with aphantasia cannot use visualization as a mode of knowledge representation or information processing. Although they can dream in images and can experience involuntary flashes of mental images in a waking state, they cannot use mental imagery deliberately. 

In this absence of phenomenal and sensory imagery [82], individuals with aphantasia display differences in cognitive processing when compared to neurotypical individuals. For example, individuals with aphantasia will not experience fear associated with reading or imagining frightening stories, but only if shown a frightening image [83]. On the other hand, when asked to report the number of windows in their house, participants would be able to draw their house to count the windows, and would report referring to a-modal ‘knowledge’, or to a ‘subvisual’ modality to do so [81]. Additionally, individuals with aphantasia display significant difficulties with autobiographical memory and face recognition [84]. Finally, it should be noted that about 50% of individuals with aphantasia will also report a poor or lacking vividness in other sensory modalities of mental representation [84,85].

These findings illustrate that vividness is neurologically plausible and moreover demonstrate more objective measures of vividness in comparison to a self-reported test [68] (again, see Figure 5). More important, neuroscientific findings make clear that depictive reasoning remains the preferred form of reasoning. Indeed, research has shown that humans attempt to represent or imagine certain situations explicitly and in too much detail [66]. Within cognitive psychology, D’Angiulli and Reeves [86] have referred to this information processing preference or tendency as the *vividness-core principle*. Although he did not call it this way, Roy [87] claimed that this information processing tendency provides an intersection where a vivid AI and a human could reason in the same manner about an environment represented in a similar way in both beings.

Thus, not only can the vivid-core principle provide a potential platform for communication among humans, but also between humans and AI. The latter synthesis could not be more fitting than in the particular field of neuromorphic AI and the development of artificial neural networks.

## 4. Vividness in Brain-Inspired AI (Deep Learning)

### 4.1. Visual Representation in Artificial Neural Networks

The human ability to form internal visual representations, as well as the ability to reflect upon them when making decisions, is one that remains a challenge to be integrated into advanced AI systems, such as artificial neural networks. As noted by Besold et al. [88], there is a current need for an integration between a symbolic and neural approach to AI, in order to be able to develop models that approach the biological and cognitive models observed in cognitive science research. On one hand, to achieve the complex symbolic processing that the human brain is capable of, it is desirable for the architecture of the AI to be based on the neuronal structure of the brain, such as neural networks. On the other hand, neural networks with neural-symbolic representation would offer various advantages in performance, learning capacity, generalization to similar input, interpretability, and extraction of knowledge [88]. 

Here, we will discuss various attempts at integrating a neural-symbolic representations in the context of vividness and AI. We will call this a ‘brain-inspired approach’ to AI, as opposed to a symbolic approach. In the context of this review, we are specifically looking at attempts where the neural networks were able to process visual representations, and then those that were also able to create an internal visual representation and used such representations to inform their decision-making processes.

One of the first examples of neural networks processing visual representations comes from the field of image recognition in AI. The ability to take images as inputs into the network is achieved by connecting a visual component to neural networks, in the form of graphics processing units (GPUs) for example, in order to produce faster and more accurate results. ImageNet competitions have sparked an interest for integrating perhaps a more “vivid” component in the architecture of convolutional neural networks, as they have been able to provide faster results, process more computations, and achieve greater precision.

An example of this that attracted notable attention from the community in artificial intelligence (AI) was AlexNet, a convolutional neural network (CNN) designed by Krizhevsky and Sutskever [89], which was able to successfully implement graphics processing units (GPUs) as part of the CNN design, in addition to introducing data augmentation, dropout, and overlapping pools of neurons in their design, in order to achieve faster date output and with higher precision. This architecture of AlexNet makes it particularly efficient for object-detection tasks, achieving top-5 error rates of 15.5% on the 2012 ImageNet Large Scale Visual Recognition Challenge competition, dethroning the previous best neural network. 

In addition to the multiple GPUs processing outputs from different sets of neurons, key features of the design that allowed to achieve such accuracy were the introduction of network design approaches that were not usual for traditional CNNs, such as dropout and overlapping. These two techniques manipulated the information integration made by the network’s neurons: (i) dropout would allow to turn off certain neurons with a set probability, in order to diversify each neuron’s ability to connect with a random neuron and still produce a robust result; (ii) overlapping would permit the CNN to retrieve output from overlapping groups of neurons, allowing for more diversified communication between the network’s neurons, a reduction in error, and a lesser tendency for overfitting. By creating a network that allowed for better and more diversified information integration, higher accuracy and processing speed was achieved. In this case, it could be said that AlexNet has been built with an architecture allowing for a greater degree of vividness.

The benefit of image recognition goes beyond simple image identification. It can have applications in facial and emotion recognition, which can be implemented in security systems or in hospital settings that would allow to replace certain patient management roles [90]. The use of neural network to produce creative works such as music [91] and paintings is also gaining popularity [92]. 

One caveat remains: Even though they use a form of visual processing, image recognition networks are simply making associations from their knowledge base to the sensory input they receive. This is not enough to indicate that there are, in fact, internal visual representations that are formed from the sensory inputs, nor that those internal representations are being manipulated by the AI to assess information or produce novel outcomes. Finally, this also does not indicate any intentionality in the systems visual ‘reasoning’, as there is no proof of intentionality in the relationships between the concepts in the VKB of such networks and their external world analogues [93]. 

### 4.2. From Vividness to Consciousness

Another way to think about vividness is the potential association between the level of internal activity required to produce increasingly vivid internal representations (as in Marks’ VVIQ-vividness), and how this type of activity could hint at sentience, or consciousness. To speak of consciousness in AI, there needs to be a way to assess the level of consciousness within a machine, which poses a certain level of difficulty when the measures of consciousness in humans are yet to be refined and certain. In such a case, could vividness—as a measure of how explicit the relations between two concepts are, or more specifically, as to how explicit the connections between neurons within a network are while leading to a certain action, thought, or emotion—provide an indication for determining the level of consciousness in an intelligent agent?

One problem that surfaces when trying to assess vividness or vivid representing in AI, and especially in ANNs, is that as the system’s architecture becomes increasingly complex, it becomes almost impossible to determine which processes lead to its outcome. This becomes an alarming problem for applications in which AI plays a role that can have significant impact on a human life, such as if an AI is trained to diagnose patients, or if it is to determine an arrested individual’s level of threat to others. It is important to understand how the AI produced false positives and false negatives, in order to correct them properly, which currently, is quite a challenging, if not impossible, task. Gamez and Aleksander [94] explained the ANN becomes analogical to a black box; only the input and the output can be observed, and only these observations can inform the adjustments to be made to the ANN’s architecture, input, or training, in order to get a more desirable outcome. 

However, this approach is not viable for increasingly complex ANNs, nor for systems where multiple ANNs collaborate to produce an outcome. There is no way to determine with precision the cause of an unexpected behavior or outcome as only external behavior can be observed, and in systems with multiple ANNs, it is difficult to determine which ANNs communicated with each other, and which tasks or computations they took part in. This is also making it quite an impossible task to determine the level of vividness of a neural network—the network of neurons that were activated, and when they were activated, and what were their weights—during its computational process leading up to an outcome. 

ANNs are, in their most basic form, inspired from the human architecture of the brain. A series of connected neurons, sometimes in multiple layers, communicate in order to produce an outcome. An agent responsible for a more complex task, such as facial recognition, would also benefit from a neurobiologically inspired architecture. According to Aleksander and Gamez [95], deriving ANNs’ architectures from a human model of cognition not only would allow for faster computation, but may also help observe the inner states of the ANN during the computational process. This ability to observe states could also help measure vividness, and a more concrete measure of vividness may become a tool to help assess levels of consciousness.

So far, we know of two algorithms that have been established and that have potential to provide insight as to how vivid is the computational process of a given ANN. One is the state-based Φ suggested by Balduzzi and Tononi [96], another is the measure of liveliness provided by Gamez and Aleksander [94].

The model introduced by Balduzzi and Tononi [96] considers that consciousness of an intelligent agent is the neural network, and the consciousness of the network can be quantified mathematically by taking into account the quality and quantity of the neurons spiking within the network. The outcome of such calculation, the state-based Φ, is what helps quantify and determine the degree of the network’s consciousness. One of the drawbacks in this proposition, however, is that this still does not address the black box issue, since it does not allow to directly observe the network’s internal states of consciousness. Although the formula for determining the levels of “consciousness” of the ANN seems to produce the promised results, it does not allow to view each state of the ANN during its computation of the outcome, nor to assess how conscious each of these stages were, or if there is one stage or layer of neurons that had a greater impact on a particular outcome than the others.

The state-based measure of liveliness presented by Gamez and Aleksander [97] is designed to address this problem. Liveliness is defined by the quantity and quality of the spiking of neurons with the neural network. However, by implementing a weight threshold for the neurons to fire, such as exists in the human model of cognition (i.e., the action potential of neurons), they are also able to identify which neuron fired and when. This allows them to assess the degree of liveliness of a network not only as a whole, but at a given state during the computational process. In this manner, the authors are addressing the black box problem of ANN they have previously described [94], while also providing a way to assess the liveliness of the ANN internal states. 

It is difficult to establish whether the state-based Φ or the state-based liveliness are accurate measures of vividness of a network. While the state-based Φ may determine areas of vividness in an ANN better than the state-based liveliness measure, it does not fare as well as the latter for interpreting larger, more complex ANNs [97]. Moreover, although a greater neural connectedness could hint at consciousness, it still does not prove the network possesses intentionality. This limitation of information integration theory becomes very evident when contrasted to the Benussi-inspired phenomenological approach to vividness we outlined earlier, which instead allows a more direct understanding of internal states of consciousness.

### 4.3. Iconic Transfer and Intentionality

A vivid knowledge representation can provide the base for an iconic state structure within an AI [98], allowing the ability to ‘think’ without the usage of words, and without having to learn a complex linguistic structure while still retaining the ability to reflect on and perform complex tasks. An iconic state structure is a form of reasoning that operates solely on visual representations, or ‘icons’, associated with objects in the world, and the various associations that can be made between these icons for input processing and production of outputs. Interestingly, iconic state structures are not too far removed from a phrase structure of processing information. 

Aleksander [98] explained that humans possess the ability to recognize the equivalence between sentences of different structure sharing the same meaning (i.e., “dog bites man” or “the man is bitten by the dog”) long before even being able to understand the complex linguistic concepts that allow for this understanding, such as *transformational grammar*, as defined by Chomsky, and *case roles*. The only way for such an ability to be inherited is to have iconic states associated with each word or concept. Thus, the argument is that iconic transfer is precisely what allows understanding links and equivalencies between concepts, without having to understanding all the complexities attached to a linguistic form of expression and information sharing. 

**Figure 7:**
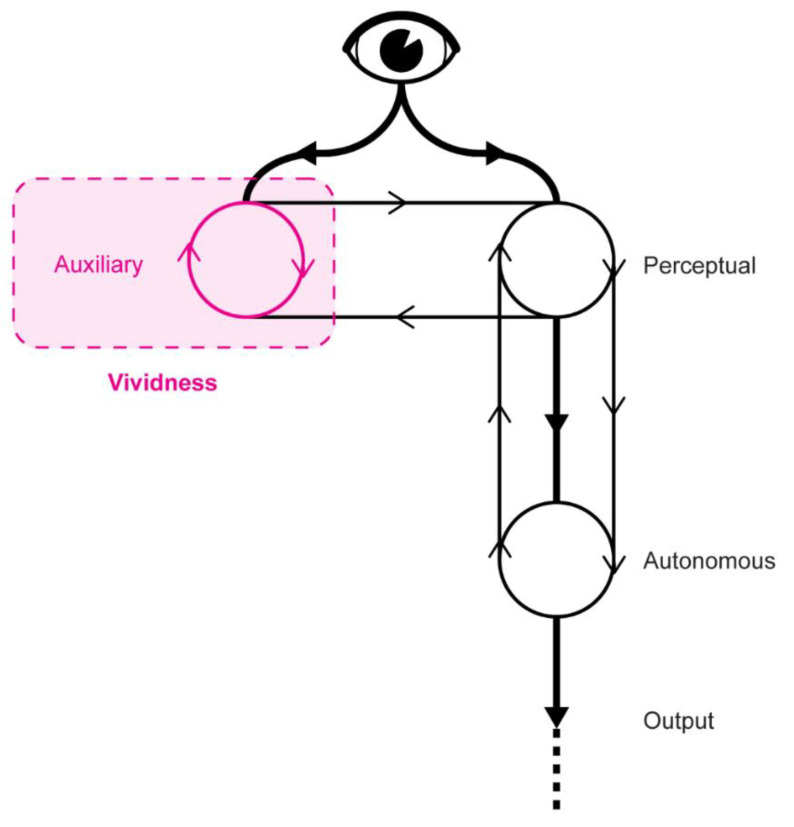
In neural networks, vividness could be conceived as arising from auxiliary processes that connect to perceptual conscious thinking processes. According to Aleksander [98], the exchange of neural signals between auxiliary and perceptual conscious processes has been identified as iconic transfers, and results in conscious sensory perception, or a machine gaining the ability to reflect on itself and its decisions. Figure adapted from Aleksander [98].

For this to be instilled in an AI, Aleksander [98] argued there would need to be an implementation of a system capable of transferring sensory signals into inner neurons. Furthermore, the author positioned that intentionality stems from this precise ability to perform iconic transfers, rather than mere attribution of concepts to objects in the world. In other words, a machine able to reflect on itself and on its environment using internal iconic states, and able to combine these to produce its own iconic states as a result (see Figure 7), is a much closer indicator of qualia, and perhaps, consciousness itself. 

One project that most closely attempts to embody an idea of intentionality, while incorporating vivid representing and iconic transfer, is Aleksander’s MAGNUS [99]. MAGNUS stands for “Multiple Automata of General Neural UnitS” and is a neural network capable of creating its own representations of the world, similarly to the human capacity to have internal visual representations of the surrounding world and its objects, and to create links between them (see Figure 7). 

The structure of MAGNUS relies on three main elements: sensory inputs, neurons, and dominant synapses. The concept of dominant synapses in this neural network is key, as they operate in a way to allow or prevent other neurons from firing given a current pattern of inputs. This is somewhat analogical to the activation step in ANNs, where the network evaluates its sum of inputs to determine its confidence about the type of output to select. In the case of MAGNUS, the dominant synapse determines whether a pattern of inputs can result in further firing of a network of neurons. This gives the network a self-regulatory ability, where it can create internal states from the input it receives, such as that sensory input A will have an internal representation A’ created by the network. This results in a series of internal representations, A’, B’, C’, etc., which can then be used to inform a decision, or to create links between those states. 

Note that a first observation here is that, unlike the case of Levesque [2] vividness, this type of neural network allows both direct representations of the world, but also the creation of new representations and links between those representations, which is an ability not usually observed in the behaviors of either symbolic programs or common neural network applications. To quote Aleksander [99]“…where a computer can be viewed as a filing cabinet (choose your file, put things in it or take things out), the neural state machine has a completely different way of achieving memory which includes a vivid pictorial memory with powers of accessing itself. This explains some of the characteristics of human memory which psychologists have identified: episodic memory (remembering events), semantic memory (remembering things which may not have been experienced) and working memory (the brief act of memory which is present during perception).”

This ability to form internal states calls for a particular type of learning—the iconic training. By this, Aleksander [99] suggested three core aspects of such a training: (1) a dominant synapse is feeding back to other neurons, (2) a temporal association, and (3) multi-input associations. While the dominant synapse allows for the formation of internal representations, temporal associations allow to create orderly and chronological orders between those representations. Meanwhile, multi-input associations are what allow the network to link concepts together that may not have been explicitly shown to be linked in the external world. Such iconic training eventually allows the network to use internal representations for processes such as simulate itself in the external world, simulate its own capabilities, and identify its own mental state or where a specific object in the world is located. 

**Figure 8:**
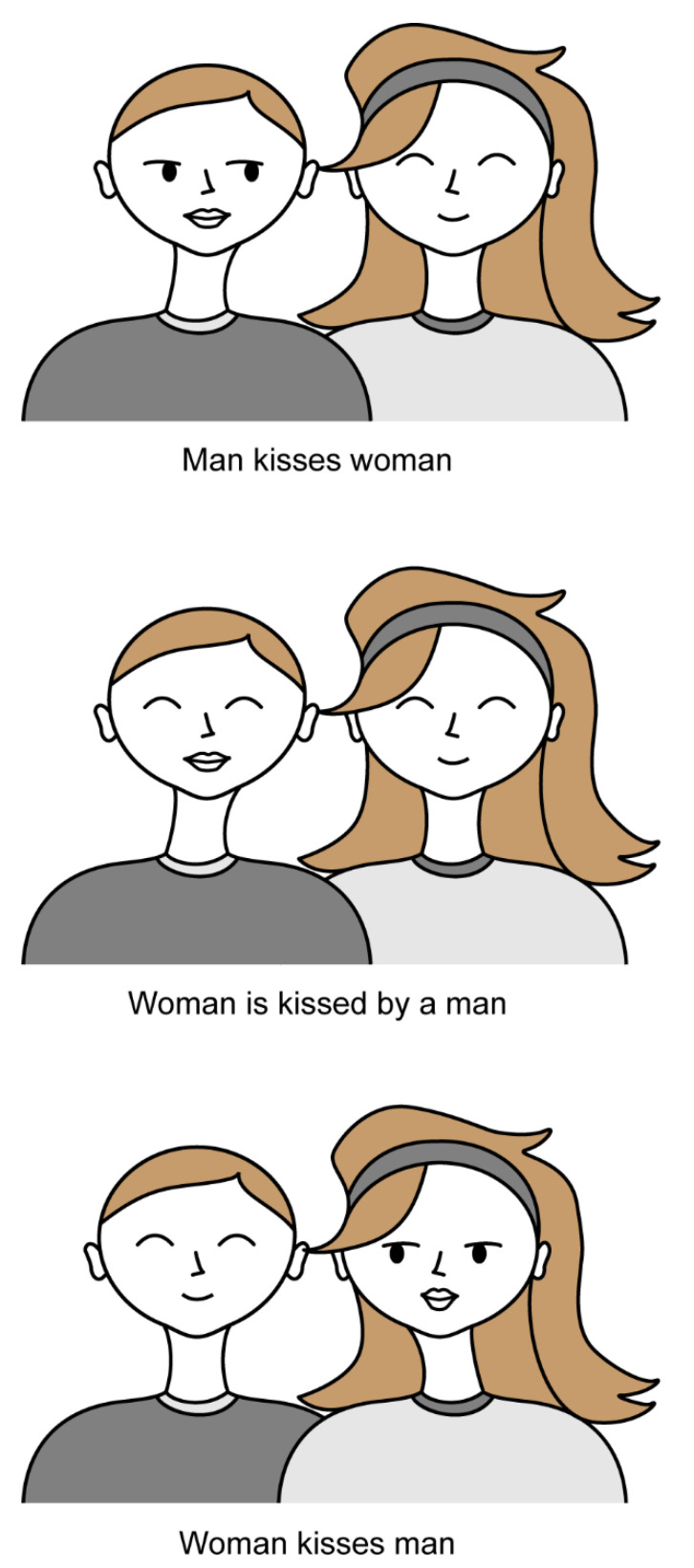
Illustrated example of an iconic syntactic structure that is created and used by MAGNUS.

As such, in one experiment, MAGNUS was presented with sequences of different parts of a portrait. The network not only successfully created internal representations for each of the parts of the image, but also internally associated each of those parts with a concept (i.e., “eye”, “nose”, “pipe”, etc.) and understood the associations between those images (i.e., “eye” is next to “nose”; “pipe” is in the “mouth”). An example of these complex iconic structures is shown in Figure 8, where a simple modification of a feature of the iconic schemas of “man” and “woman” can be used to signify the action or verb and constrain the subject-verb-object sentence or iconic scene and its meaning.

Importantly, the example of Figure 8 also suggests the possibility that network could use the structure to generalize and represent a new “vivid” state of events, namely, “woman and man kiss each other”. Again, this demonstrates the ability of the network to autonomously create and use associations between internal states, without being explicitly prompted to do so by its environment, thus hinting at an idea of intentionality, both as the author of MAGNUS describes it [98] and fitting Searle’s interpretation of intentionality at once [93].

### 4.4. Future Directions

To demonstrate theory in practice, there is perhaps an existing example of a robot that may be exhibiting the capabilities of MAGNUS. Boston Dynamics have created Spot, a robot capable of assessing its environment, identifying objects, assessing whether they can interact with these objects, and executing an interaction where they continuously monitor the execution and outcomes of the action. 

For example, if Spot circulates in a room and identifies a door, it will further analyze the door and identify a handle, which will prompt Spot to deploy a robotic arm attached to its body to open the door. Spot will adjust its own position and the pulling and clenching of the door’s handle, depending on how heavy the door is, and whether something is blocking the door or pushing it back to a closed state [100]. Furthermore, once a door is open, it can choose to leave the door open to let another Spot walk through, before walking through the door itself, by continuously keeping the door open and out of its own way. 

The series of actions and continuous self-monitoring of Spot demonstrate a potential example of a robot that can, just like MAGNUS, simulate itself and others in the world to assess its capabilities (i.e., the door needs to be opened so that the Spot itself or another Spot can pass), that potentially possesses the ability to identify its own internal mental states (i.e., the door requires more force to be fully open, Spot assesses the shift in force and weight of the door on itself and adjusts the position of its arm and body to open the door), and the ability to assess where an object in the world is located (i.e., Spot is able to identify a door, or where to direct itself). It leaves to wonder whether Spot actually does develop internal representations of the world in order to achieve such complex tasks, and whether it is able to develop novel links between previously unrelated internal representations or even to create new, ‘imagined’, internal representations about the world that inform its actions, hinting at possible intentionality. 

Other than opening doors, the robot’s capabilities are currently explored to serve as a maintenance staff for water dam facilities [101]. This task requires Spot to autonomously orient itself in various environments of such facilities, autonomously direct itself through corridors and steep staircases to proper amenities to be analyzed for functionality, and, finally, to be able to identify anomalies on these amenities and send an alert to a human engineer. Furthermore, Spot can be seen interacting with live organisms, such as a dog [102], and choosing to either avoid the dog, ‘observe’ the dog, or produce agitated movements with its robotic legs, which seem to simulate the excitement and playfulness of a dog. Are the tasks that Spot is able to accomplish simple attributions between a sensory stimulus and an internal concept, resulting in an action, or do they require the production of internal representations? Does Spot use these internal representations to choose its next actions? Those questions matter, as this ability to reflect upon itself points to a level of self-awareness, and an AI possessing self-awareness, all the while being capable of doing certain tasks much better than humans, could eventually become beneficial beyond measure, …or a looming threat. 

### 4.5. Ethical Caveats

Assuming that in one way or another we are able to prove, without a doubt, that AI systems such as those built in Spot or MAGNUS truly possess a particular level of vivid representing, consciousness, intentionality, and thus a level of sentience and self-awareness, it would be important to reflect on the outcomes and possible consequences that this could entail. Even with the question of inner iconic states and consciousness aside, a number of concerns with the evolution of the AI technology are already being pointed out by authors such as Amodei and Olah [103]. Safety of continuing AI production without proper understanding of what that AI is and is capable of doing can lead to AI behavior that can be potentially harmful to humans [103].

As one of the ways to prepare appropriately for such problems, it is important to understand vividness under a scope larger than its initial theoretical definition given by Levesque [2]. As Irving and Askell [104] suggested, if we create AI to serve humans, we also need to take the time to appropriately understand humans first, their psychology, their neurobiology, and the way they are socially, and philosophically (as in, do humans have a consciousness, to what level and how to identify it?). Several organizations, such as OpenAI, DeepMind, Berkley’s CHAI, and the AI safety organization Ought, have already begun investigating questions of AI safety, for example with regards to iterated amplification in AI and in humans.

## 5. Conclusions

In this paper, we first reviewed attempts at defining a symbolic approach to vividness, with major contributions from the works of Levesque [2,6,7] that defined vivid knowledge representation in AI requires at least two main characteristics: (1) the VKB is ‘complete’ as there is a direct correspondence between objects in the world and concepts in the VKB, as no uncertainties can be permitted; (2) the VKB is ‘true’, insofar as the associations existing between object in the world also exist in between the corresponding concepts in the VKB. In response, various authors pointed to weaknesses of abiding by those principles. For example, a complete VKB renders it difficult to store knowledge that is changing over time, or knowledge that is partial and contains some unknowns. However, allowing for unknowns would give more potential to the VKB to solve more complex problems. 

In parallel, we reviewed the perspective on vividness from the lens of cognitive science research. We established that vividness as a phenomenological phenomenon is plausible and there are psychological and neurological differences that distinguish individuals reporting acute inner imagery from those who do not, and from those who lack inner mental imagery. We also reviewed that inner mental representations act as an additional form of database or memory that individuals can access and reflect upon to inform their decisions.

Finally, we reviewed a neural network approach to vividness, and attempts at integrating VKB with knowledge from cognitive sciences to create an AI. Attempts such as Aleksander’s MAGNUS point to a technology with particularly complex vividness, one that approaches sentience and forces ourselves to ask the question “What does it take for a robot to be deemed conscious?”. On the other hand, creations such as Spot (and the other robots) which directly interact with individuals give rise to ethical questions and concerns: “Are these robots conscious? If they are, what does that mean for their rights? What does that mean for us and our safety?”.

Certainly, as our technological needs continue to advance at rapid pace, it is important to deepen our understanding of human and AI cognition alike, in order to create AI with an architecture that allows solving complex tasks, which is hardly possible without a system allowing to glance at the internal states of processing, which in turn can hardly be created without a vivid knowledge base and iconic transfer. However, adding this type of complexity comes with concerns for ethics and safety: if such a system is capable of vividness to the point of having or developing a consciousness and intentionality, this means that on one hand, we may need to develop regulations on treatment of AI, but also regulations on how to build AI that remains safe for humans, in both short and long term. This cannot be done without a unification of sciences, that is, without developing a better understanding of concepts such as vividness, intentionality, and consciousness, through the unification of cognitive sciences with AI. Developing this unified understanding of human and AI cognition constitutes an important, yet still under-researched, avenue of exploration. As Goebel aptly argued: “Ultimately, the human is never extricated from the computational framework, so the desire for programming and problem solving efficiency is always implicitly connected to human cognition” [64]. To conclude accordingly, a unitary notion of vividness as discussed in this paper may perhaps offer a viable lingua franca for psychologically-plausible simulation and modeling across all disciplines dealing with reasoning and knowledge representation.

## Data Availability

Data sharing not applicable.
